# Supplementary material for: Patterns of Intron Gain and Loss in Fungi
Source: PLoS Biol. 2004 Nov 30;2(12):e422. doi: 10.1371/journal.pbio.0020422 (PMC532390; doi:10.1371/journal.pbio.0020422)
Supplement: Table S1 — Also available at http://genes.mit.edu/NielsenEtAl/. (4.3 MB ZIP). [file pbio.0020422.st001.zip › NielsenEtAl/html/1226.html]

AN2306.1.NCU06606.1.MG05093.1.FG05251.1


```
 CLUSTAL W (1.82) Multiple Sequence Alignments - Introns Inserted


Sequence 1: NCU06606.1	231 aa
Sequence 2: MG05093.1	236 aa
Sequence 3: FG05251.1	235 aa
Sequence 4: AN2306.1	238 aa
Alignment Length: 249 aa
Number Identitical Residues: 126 aa
Alignment Score (without introns) 5975


MG05093.1 	MAPLTTATRAITRCAASPSKAVTTAARALSTTPAQNGSSGAS-FSSPFR--GEQKSTQIP
NCU06606.1	MAPVSIVSRAAMRAAAAPARAV----RALTTSTALQGSSSST-FESPFK--GESKAAKVP
FG05251.1 	MATLATASRLCLRAAAKPVAPA---VRALSTTAIRPDSASASGYSSPFKFQGESKGSQIP
AN2306.1  	-MALSFTSGSLLRACARKQLST------TSRAVVASQQRYSSSFDSPFT---AKDTLKVP
          	  .:: .:    *..*    ..       : :     .  ::.:.***     ..  ::*

MG05093.1 	DFKKYMAPKGSSTSNAVFSYFMVGAMGAISAAGAKSTVQE1FLVNMSASADVLAMAKVEV
NCU06606.1	DFGKYMS-KAPPSTNMLFSYFMVGTMGAITAAGAKSTIQE1FLKNMSASADVLAMAKVEV
FG05251.1 	DFGKYVS-TGSEGKNKLYSYFMVGALGAVSAAGAKSTVQE1FLVNMSASADVLAMAKVEV
AN2306.1  	DFSKYSS-KKAPRSNQVFSYFVAGTMGLASAVGAKATVQD1FLVNMSASADVLAQAKVEI
          	** ** : . .  .* ::***:.*::*  :*.***:*:*: ** ********** ****:

MG05093.1 	DLTTIPEGKN0----------VIIKWRGKPVFIRHRTGREIDEANKIDVASLRDPEADSD
NCU06606.1	DLNAIPEGKN0----------VIIKWRGKPVFIRHRTPAEIEEANKVNVATLRDPETDAD
FG05251.1 	DLASIPEGKN0----------VIIKWRGKPVFIRHRTQDEIDQANKVNISSLRDPEEDSA
AN2306.1  	GLATIPEGKN0ALRLNRSFQQVIIKWRGKPVFIRHRTQDEIKEAQEYDWKTLRDPQPDEE
          	.* :****** :   . : ..****************  **.:*:: :  :****: *  

MG05093.1 	RVKKPEWLVMLG1VCTHLGCVPIGEAGDFGGWFCPCHGSHYDISGRIRKGPAPLNLEIPA
NCU06606.1	RVKKPEWLVMLG1VCTHLGCVPIGEAGDYGGWFCPCHGSHYDISGRIRKGPAPLNLEIPL
FG05251.1 	RARQPEWLVMLG1VCTHLGCVPIGEAGDYGGWFCPCHGSHYDISGRIRKGPAPLNLEIPE
AN2306.1  	RVQKAEWLVMLG1VCTHLGCVPIGESGDFGGWFCPCHGSHYDISGRIRKGPAPLNLEVPQ
          	*.::.******* ************:**:****************************:* 

MG05093.1 	YDFPEDDKLVIG
NCU06606.1	YEFPEEGKLVIG
FG05251.1 	YDFPEEGKLIIG
AN2306.1  	YNFPDEETLIIG
          	*:**:: .*:**
```
